# The need of preexposure prophylaxis against COVID-19 in immunocompromised patients– an assessment from Germany

**DOI:** 10.1007/s15010-025-02540-w

**Published:** 2025-04-22

**Authors:** Jacob Gerstenberg, Christoph Lübbert, Marek Widera, Benjamin T. Schleenvoigt

**Affiliations:** 1 Institute for Tropical Medicine, Eberhard-Karls University Tübingen, Tübingen, Germany; 2 Department of Internal Medicine, DIAKOVERE Friederikenstift, Hannover, Germany; 3 Division of Infectious Diseases and Tropical Medicine, Department of Medicine I, Leipzig University Medical Center, Leipzig, Germany; 4 Institute for Medical Virology, Goethe University, University Hospital Frankfurt, Frankfurt am Main, Germany; 5 Institute of Infectious Diseases and Infection Control, Jena University Hospital, Friedrich-Schiller- University, Jena, Germany

With great interest we read the work of Haidar et al. “Efficacy and safety of sipavibart for prevention of COVID-19 in individuals who are immunocompromised (SUPERNOVA): a randomized, controlled, double-blind, phase 3 trial” [1]. This prospective study investigated the protective effect of the new monoclonal antibody (mAb) sipavibart (formerly AZD3152) as pre-exposure prophylaxis (PrEP) in n = 3349 immunocompromised patients. Overall, the authors report a significant risk reduction of 34.9% (97.5% CI 15.0-50.1, p = 0.0006) for symptomatic COVID-19 over a period of 6 months.

Prevalence of immunosuppression depends on the underlying definition used and can be as high as 10% if chronic liver-, kidney-, and lung diseases are also considered [2]. Meanwhile, more conservative estimates focusing only on severe immunosuppression, such as those caused by allogeneic stem cell transplantation, solid organ transplantation or B-cell depletion, may better depict those populations with the most urgent need for prophylactic treatment with mAbs. Reasonable estimates of this population that have been reported lay between 4 and 6% [2, 3]. In the current winter season, there have been 129,000 notified COVID-19 cases and more than 48,000 (38%) associated hospitalizations in Germany (as of March 2, 2025), but patients with IS are not separately recorded in the reporting system [4]. In the UK, the proportion of patients hospitalized due to COVID-19 with underlying IS is reported to be 21% [5]. To avoid such hospitalizations, effective protection strategies are needed, especially when active vaccination against COVID-19 fails.

The ongoing challenge of researching mAbs against COVID-19 lies in the rapid, unpredictable emergence of resistance. In the SUPERNOVA study, the resistance-associated mutation (RAM) F456L occurred in 47/122 (38.5%) of SARS-CoV-2 detections. Jian et al. previously described this RAM in 2023 in combination with RAM L455F, which alone led to a limited loss of efficacy of the sipavibart precursor Omi-42 [6]. Together, however, L455F and F456L synergistically enhanced antibody escape and ACE2 binding, resulting in a dramatic loss of neutralization efficiency in vitro. SUPERNOVA showed a significant risk reduction of 42.9% without RAM F456L (compared to an insignificant 30.4% for the F456L subgroup) within 6 months, consistent with the observations of Jian et al. Although individual mutations like F456L can severely impair AZD3152 binding and virus neutralization, other mutations, including T415I and K458E, identified through in vitro passaging of recombinant SARS-CoV-2 XBB.1.5 under increasing mAb concentrations, may function as resistance-associated mutations (RAMs) and contribute to resistance [7]. This questions which other RAMs may have occurred in the sequenced virus samples during the SUPERNOVA study. Additionally, comparison of mutations between sipavibart-treated individuals and control groups could reveal clinically relevant escape mutations, particularly since viral evolution in immunocompromised hosts may differ from that in healthy individuals and in vitro.

Another point of criticism is the heterogeneity of the control group: out of 1675 participants, 1102 received tixagevimab-cilgavimab (the previous mAb with assumed ineffectiveness), while only 561 received placebo. We therefore propose a subset analysis to separately evaluate the study group against both the tixagevimab-cilgavimab and placebo groups. This would help to exclude residual efficacy of tixagevimab-cilgavimab and to assess a potentially higher protective effect of sipavibart compared to placebo, if the possibly low number of endpoints achieved in subgroups allows such an analysis.

## Data Availability

No datasets were generated or analysed during the current study.
